# A New Representative Sampling Method for Series Size Rock Joint Surfaces

**DOI:** 10.1038/s41598-020-66047-0

**Published:** 2020-06-04

**Authors:** Man Huang, Chenjie Hong, Chengrong Ma, Zhanyou Luo, Shigui Du, Fei Yang

**Affiliations:** 1 Department of Civil Engineering, Shaoxing University, 508 Huancheng West Road, Shaoxing, 312000 Zhejiang China; 2 Department of Civil and Environmental Engineering, Louisiana State University, 3255 Patrick F. Taylor hall, Baton Rouge, Louisiana 70803 USA; 3 Geotechnical Engineering Institute, Zhejiang University of Science and Technology, 318 Liuhe Road, Hangzhou, 310023 Zhejiang China

**Keywords:** Petrology, Geomorphology

## Abstract

The greatest variability in both shear strength and roughness exists for joint samples with smaller size, which underscores the necessity of performing representative sampling. This study aims to provide a representative sampling method for series size joint surfaces. The progressive coverage statistical method is introduced to provide the sufficient sample capacity for series sampling sizes by setting different propulsion spaces. The statistical law of the joint surface morphology at different sampling sizes is measured by the 3D roughness parameter with ${{\theta }}_{\max }^{\ast }/({C}+1)$. Through an application in nine natural large-scale rock joints, nine consecutive sampling sizes from 100 mm × 100 mm to 900 mm × 900 mm are selected and 121 samples are successfully acquired from each sampling size. According to the frequency distribution of roughness statistics, a new sampling method combining the layering principle and K-medoids clustering algorithm is proposed to screen representative joint samples for each sampling size. The sampling results that meet the test accuracy requirements suggest the possibility of realizing an intelligent sampling method. In addition, the representative of the interlayer cluster center is validated. Finally, the comparison results with the traditional stratified sampling method prove that the proposed method has better stability.

## Introduction

The shear behavior of rock joints is largely determined by size^1,2^. Hence, numerous shear test investigations on the scale effect in rock joints have been conducted^3–7^. Such investigations usually divide a large natural or artificially reproduced rock joint specimen into various joint models with small sizes. Direct shear tests under a given normal stress are then conducted on these joint surfaces of series sizes. Finally, the scale effect on the peak shear strength is obtained by comparing the average peak shear strength of the rock joints of each specimen size to that of the original specimen^8^. In this process, the material properties of different-sized joint specimens are the same in those of the original specimen. Therefore, the acquisition of each specimen size is equal to obtain the surface morphology (roughness) of the specimen at this size.

Roughness parameterization provides methods for quantifying the characterization of joint surface morphology, including empirical^9^, statistical^10,11^, and fractal methods^12–14^. Then, the scale effects of joint roughness and shear strength are established^15–17^. However, the representativeness of different-sized specimens is often neglected when studying the scale dependency of the two. The mechanical test of joint models with different sizes requires that each specimen represents the undulating characteristics of a specific size. However, the results of scale effect obtained by selecting a single sample to replace all specimens for experimental studies are questionable. Therefore, the representativeness of all specimens should be systematically analyzed.

Recently, some scholars have put forward methods for the statistics of joint samples. Yong *et al*. proposed a method for obtaining continuously sized joint profiles with overlapping length^18^. Considering that the statistical method of joint samples around two-dimensional profile is noncomprehensive, Huang *et al*. proposed a progressive coverage statistical method based on the idea of overlapping sampling, which can realize the statistics of three-dimensional (3D) joint morphology in series size^19^. However, the representative sampling of joint samples is not involved.

To explore the most representative roughness samples on a joint surface, Huang *et al*. first proposed a stratified sampling method for the representative sampling of joint samples^20^. However, a large number of probability calculations limit the use of this methods as the scale of joint research increases. In recent years, clustering, which plays an important role in exploring data, has been used in the traditional joint roughness analysis^21–23^. Among them, the K-medoids clustering algorithm is widely recognized for its insensitivity to processing data outliers^24^. Therefore, the stratified sampling method may be optimized by the K-medoids clustering algorithm.

In this study, we investigate the traditional sampling methods in rock joints and propose an improved stratified sampling method, which can be combined with K-medoids clustering algorithm to perform intelligent sampling. The progressive coverage statistical method is introduced to obtain series size joint samples. Furthermore, the representative assessment of sampling results with the proposed method is tested with natural rock joints, and its feature and advantages are compared with those of the traditional stratified sampling method. In addition, the representative verification of a cluster center and sensitivity analysis of k-value in K-medoids sampling process is discussed in this paper. In doing so, the accuracy of the mechanical test is validated, and the work efficiency is improved.

## Methodology

### Traditional sampling methods in rock joints

The mechanical test of joint models of different sizes requires small-scale model specimens to be included in large-scale ones^20^. Four existing sampling methods follow the above requirements, namely, simple random sampling method, processive magnifying sampling method, equal-partition sampling method, and stratified sampling method.

1. Simple random sampling method. Considering the difficulty of performing roughness measurements in field rough joints, samples of different sizes are often arbitrarily taken from the original surface. The locations of different-sized samples primarily rely on the personal judgment and choice of researchers. Therefore, representing the corresponding size of the surface morphology with such random and irregular joint samples is not comprehensive.
2. Processive magnifying sampling method^18^. The processive magnifying sampling method refers to the sampling process in which the large-sized samples are obtained through a regular amplification of small-sized samples from a side or middle section (Fig. 1), which can overcome the irregularity of the simple random sampling method. This sampling method has been widely used in scale effect research on rock joints^25–28^. However, this sampling method is still a one-sample characterization method, the number of samples in each sampling size is small and their representativeness are unclear.

**Figure 1:**
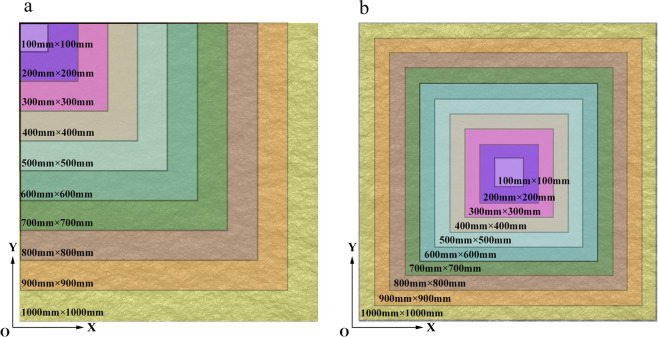
Processive magnifying sampling method. (**a**) Side amplification; (**b**) middle amplification.
3. Equal-partition sampling method^18^. To obtain different positions of the original samples, the equal-partition sampling method is applied to joint sampling^3,6^, which provide profile samples through equivalence partitioning (Fig. 2a). On the basis of this definition, the 3D surface morphology may be evenly divided, as shown in Fig. 2b. Here, it is found that too many samples of small size and too few at the large size would be provided with the equal-partition sampling method. Moreover, this sampling method cannot guarantee full coverage samples for each sampling size because some sampling sizes cannot be divisible.

**Figure 2:**
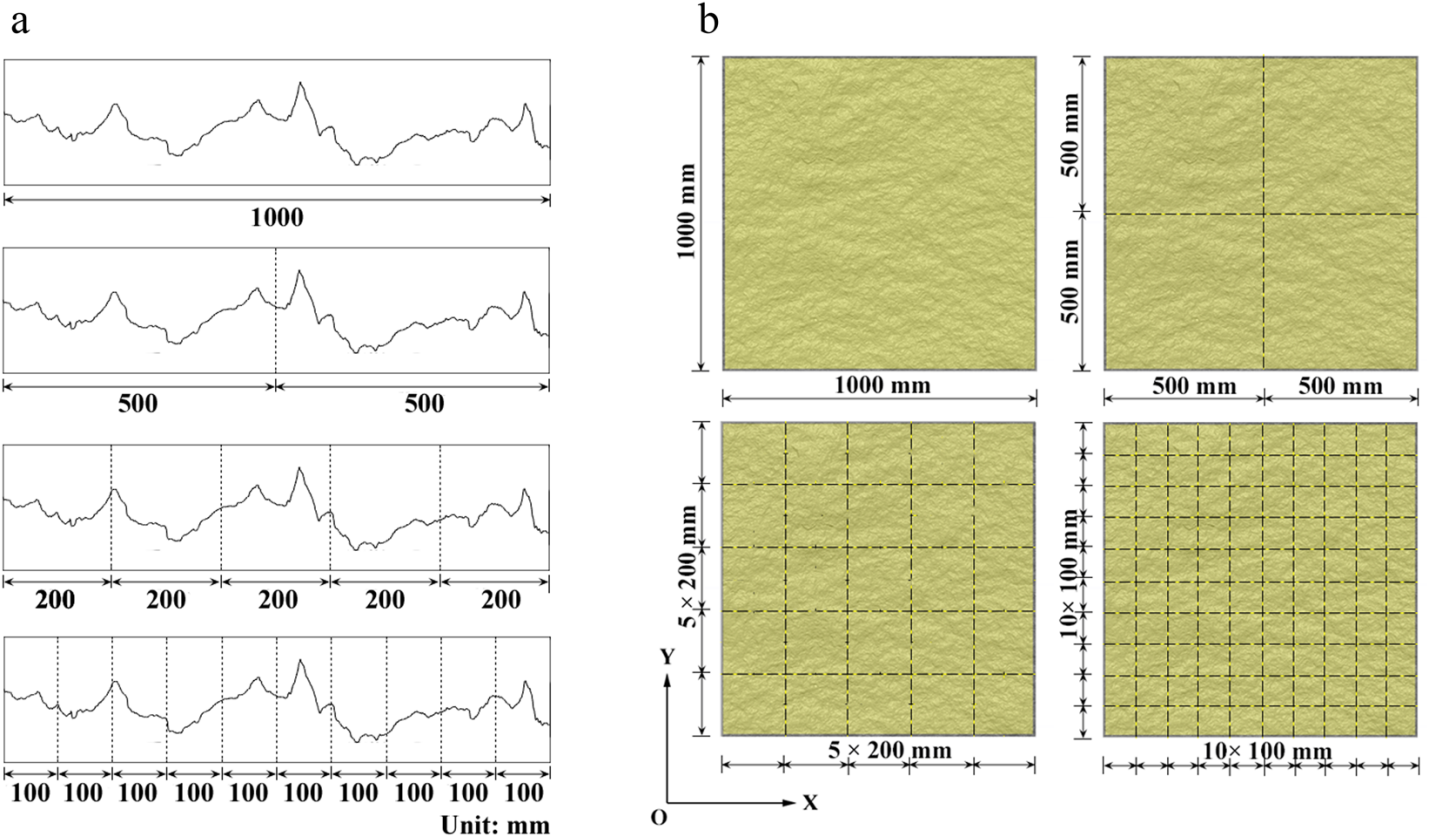
Equal-partition sampling method. (**a**) schematic diagram of uniform profile; (**b**) equal partition of the surface.
4. Stratified sampling method. The stratified sampling method introduced by Huang *et al*. is used to improve the case of having excessive samples in the small sampling size^20^. This method considers two key factors: stratified proportions are determined through the quartile method of JRC and sampling capacity is calculated within the permissible error range. The representative joint samples are then selected in the respective layer with the product of the sample quantity and stratified proportions, which will minimize the sample size and make them reasonably distributed (Fig. 3). However, due to the insufficient acquisition of large-sized sample sizes in current sample statistical methods, this method is limited to the application of small size representative samples. In addition, this method does not define the value of the interlayer samples. Hence, the arbitrarily selected joint samples may be less representative.

**Figure 3:**
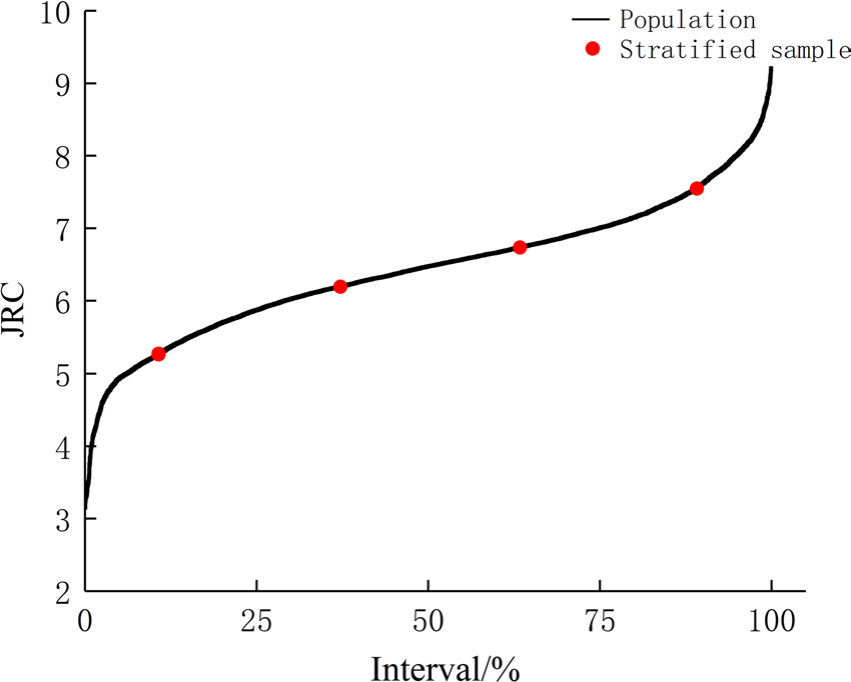
Stratified sampling method.

In summary, there are still two major shortcomings of the conventional sampling method. The first is uneven sampling (i.e., too many samples of small size and too few at the large size). The second is that the representativeness of the obtained samples is not clear. Therefore, establishing a new sampling method that can ensure the equal number of samples for different sampling sizes and therefrom select the representative samples is necessary.

### Progressive coverage statistical method

To provide a comprehensive sampling basis for the sample statistics of series sample sizes, Huang *et al*. proposed a new progressive coverage statistical method^19^. Through this method, unit samples of different sizes are propelled in orthogonal directions along the entire rock joint with different propulsion spaces (see Supplementary Fig. S1). Here, with the propulsion of the unit sample, more complete unit samples are obtained, which can cover the partial missing area morphology, increase the sample capacity, and improve the accuracy of the overall samples. Therefore, this statistical method can effectively solve the problem of excessive or insufficient samples in different sampling sizes.

### K-medoids clustering algorithm

**Figure 4:**
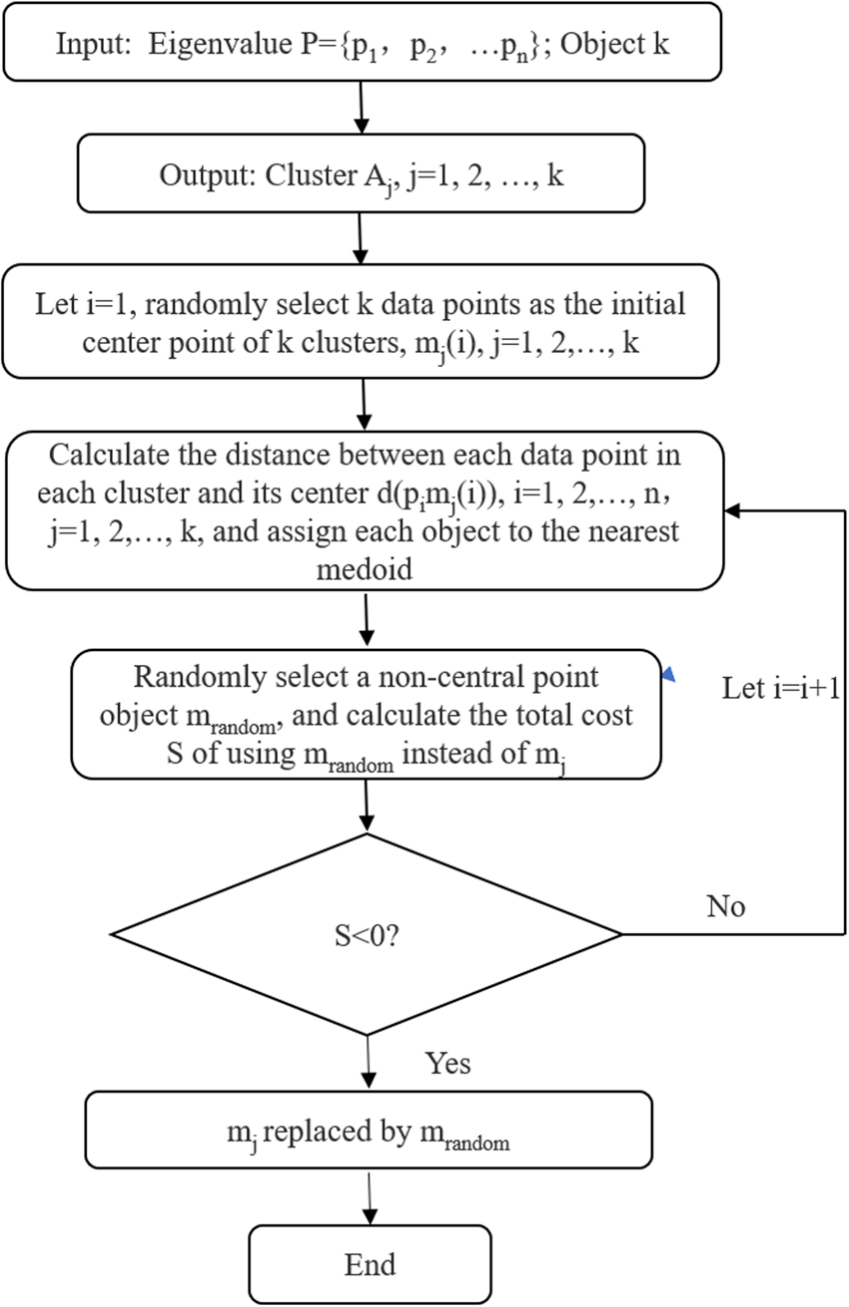
K-medoids clustering algorithm flowchart.

K-medoids clustering algorithm is a data mining tool whose aim is to find K representative objects from the data set in such a way that the sum of the within-cluster dissimilarities is minimized^29^. It assigns every object to the nearest centroid by calculating the Euclidean distance, and the expression is given by Park *et al*. as

1 $${{\rm{d}}}_{ij}=\sqrt{\mathop{\sum }\limits_{{\rm{a}}=1}^{p}{({{\rm{X}}}_{i{\rm{a}}}-{{\rm{X}}}_{j{\rm{a}}})}^{2}}\,i=1,\ldots ,m;\,j=1,\ldots ,m,$$

where *m* is the total objects, *p* is the number of variables, and *X*_*ia*_ and *X*_*ja*_ are the *a*th variable of objects *i* and *j*, respectively^24^. Figure 4 depicts the basic operation process of this clustering algorithm. The flowchart shows that the K-medoids clustering algorithm has a classification function similar to that of the stratified sampling method. Moreover, all the obtained cluster centers are real data and the most representative sample in each cluster, which means that the defect of random selection in interlayer samples is overcome. Therefore, the K-medoids clustering algorithm can be combined with the stratified sampling method for the representative sampling of joint surface morphology.

### New combined sampling method

The progressive coverage statistical method can provide enough sample size for series sampling sizes and the stratified sampling method with K-medoids clustering algorithm has great advantage in representative sampling. Therefore, we combine the two methods for the representative sampling of rock joints. To characterize the surface morphology of the joint samples, the quantitative parameters need to be determined first. In the current study of roughness parameters, Grasselli *et al*. established a relationship between total potential contact area ratio ${A}_{{\theta }^{\ast }}$ and apparent dip angle ${\theta }^{\ast }$ as follows^3^:

2 $${A}_{{\theta }^{\ast }}={A}_{0}{\left(\frac{{\theta }_{\max }^{\ast }-{\theta }^{\ast }}{{\theta }_{\max }^{\ast }}\right)}^{C},$$

where ${A}_{0}$ is the maximum potential contact area ratio, ${\theta }_{\max }^{\ast }$ is the maximum apparent dip angle in the shear direction, and *C* is the roughness fitting coefficient. Then, Tatone and Grasselli^11^ proposed a 3D roughness parameter ${\theta }_{\max }^{\ast }/(C+1)$ to evaluate the rock joint roughness, which is widely recognized^4^. Therefore, we take ${\theta }_{\max }^{\ast }/(C+1)$ as the quantitative parameters of the sample for the combined sampling method. The specific process is composed of the following steps:

Step 1: Sample statistics. The corresponding propulsion space $\Delta d$ for different sampling sizes is determined, and then the square sampling unit is progressively advanced along the surface morphology of the joints to derive the appropriate sample capacity. The sample capacity $N$ is calculated as

3 $$N={\left[\frac{L-l}{\Delta d}+1\right]}^{2}$$

where *L* is the side length of the original square joint and *l* is the side length of the target sample size, such that $l\le L$. Then, the 3D roughness parameters ${\theta }_{\max }^{\ast }/(C+1)$ of each joint sample at a given shear direction are calculated.

Step 2: Sample stratification. In accordance with the definition of the stratified sampling method, ${\theta }_{\max }^{\ast }/(C+1)$ of each sampling size is arranged from small to large, and the sample layer division is defined by the relative range into two cases. When the relative range is greater than 10%, the quartile is used as the boundary to divided all the statistical values into three intervals, that is, 0–25%, 25–75%, and 75–100%, in which the distribution proportion W of samples in each layer is 1/4, 1/2, and 1/4, respectively; and when the relative range is less than 10%, an interval of 0%–100% is defined for the statistical values because of the small variation in the sample roughness, and its distribution proportion W is 1. The sampling quantity *n* is calculated through the stratified sampling equation as follows:

4 $${\rm{n}}=\frac{\sum {W}_{{\rm{h}}}{S}_{{\rm{h}}}^{2}}{V+\sum {W}_{{\rm{h}}}{S}_{{\rm{h}}}^{2}/N},$$

5 $$V={\left(\frac{\gamma \overline{Y}}{t}\right)}^{2},$$

where *h* is the layer number, *S*^2^ is the variance, *V* is the mean variance, *t* is the upper quantile of the standard normal distribution, $\gamma$ is the permissible error, $\overline{Y}$ is the population mean, and *N* is the total sample number.

Step 3: Representative sampling with the K-medoids clustering algorithm. For one sampling size, the eigenvalue $P$ of the K-medoids clustering algorithm for the stratified samples is determined as follows:

6 $${P}_{{\rm{h}}}=\{({x}_{{\rm{h1}}},l),({x}_{{\rm{h2}}},l),\ldots ,({x}_{{\rm{ht}}},l)\},$$

where ${x}_{ht}$ represents the ${\theta }_{\max }^{\ast }/(C+1)$ values of the *t*th joint sample in *h* layer. The k-value in the K-medoids clustering algorithm is determined based on the results of the sample distribution of each layer as

7 $${K}_{h}=\lceil n\times {W}_{h}\rceil .$$

Then, the K-medoids clustering algorithm is run to obtain the K cluster centers from the data of the eigenvalue. The joint samples corresponding to these center points are the representative samples of the corresponding sampling size.

To verify the feasibility of the new sampling method, we will use it to perform the sampling in natural rock joints. In this method, the different propulsion spaces will be selected to obtain a sufficient sample size for series sampling sizes and the k-values in different layers for each sampling size will be determined to carry out representative sampling. Finally, the method evaluates the representativeness of the sampling results to show good applicability of the combined sampling method in the rock joints.

## Application

### Acquisition of a large-scale joint

**Figure 5:**
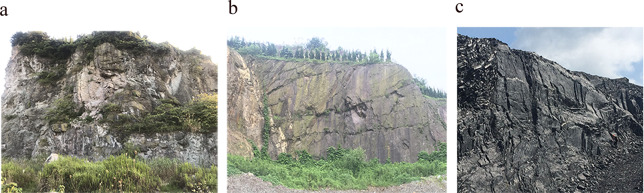
Large-scale natural rock joint. (**a**) tuff; (**b**) sandstone; (**c**) limestone.

A large-scale and well-preserved natural rock joint should be prepared before conducting the statistics of the joint samples. After many field investigations, we found three sets of different lithological joints (tuff, sandstone, and limestone), whose width and height are all more than 1 m (Fig. 5). To collect the 3D surface information, a portable laser scanner (MetraSCAN 3D, Creaform, Canada) with a scanning accuracy of 0.5 mm is used to scan the field site. Three 1000 mm × 1000 mm digitized surfaces are selected for each lithology as the original joints, labeled as T1, T2, T3, S1, S2, S3, L1, L2, and L3 (see Supplementary Fig. S2).

### Progressive coverage statistical procedure

To make a comprehensive division of rock joints, nine consecutive sampling sizes with a dimension of 100 mm × 100 mm to 900 mm × 900 mm are selected in this work. Then, the progressive coverage statistical method is used to set the propulsion spacing to 90, 80, 70, 60, 50, 40, 30, 20, and 10 mm, which can achieve 121 samples per sampling size. Hence, the progressive coverage statistical method can create conditions for the representative sampling of large-sized samples.

## Results

### Joint sample roughness distribution

Roughness measurement is performed on the obtained joint samples by means of roughness parameter ${\theta }_{\max }^{\ast }/(C+1)$ along shear direction at 0°, in which the roughness statistics of T1, S1, and L1 joints are given in the form of histograms, as shown in Supplementary Fig. S3. It shows that the distribution in roughness is quite wide at small sample sizes, but quite narrow at the larger sample sizes. This means that the smaller the sampling size, the greater dispersion of joint sample roughness. Therefore, the representativeness of the joint samples is very important. In addition, from the perspective of frequency distribution of the joint sample roughness, although the normal, skewed distribution, and irregular distributions appeared, the roughness is still distributed throughout the different intervals. This finding indicates that such data sets are suitable for screening representative samples with the stratified sampling method.

### Determination of the sampling quantity

According to the sample stratification (step 2) in Section 2, the permissible error γ is set as 0.15, and the upper standard quantile *t* in the corresponding standard normal distribution table is 1.96 when the confidence level is 95%. The interlayer variance (${S}_{0-25}^{2}$, ${S}_{25-75}^{2}$, ${S}_{75-100}^{2}$, or ${S}_{0-100}^{2}$) of each sample size is calculated and the calculated data is substituted into Eq. (4) to obtain the sampling quantity *n* and the k-values in accordance with Eq. (7). The calculation results of the T1, S1, and L1 joints can be found as Supplementary Table S1. The sample capacity of different sampling sizes gradually decreases due to the decrease in the difference between the joint sample surface morphology as the sampling size increases.

### Sampling with the K-medoids clustering algorithm

The sampling work starts by determining the k-value for the interlayer sample in each sampling size. After acquiring the eigenvalue of the samples for each sampling size, we sequentially input the eigenvalue and corresponding k-value into the K-medoids clustering algorithm. The clustering results under different sampling sizes are shown in Supplementary Fig. S4, where the cluster centers are distributed in different sampling intervals.

### Representative assessment of the sampling results

**Figure 6:**
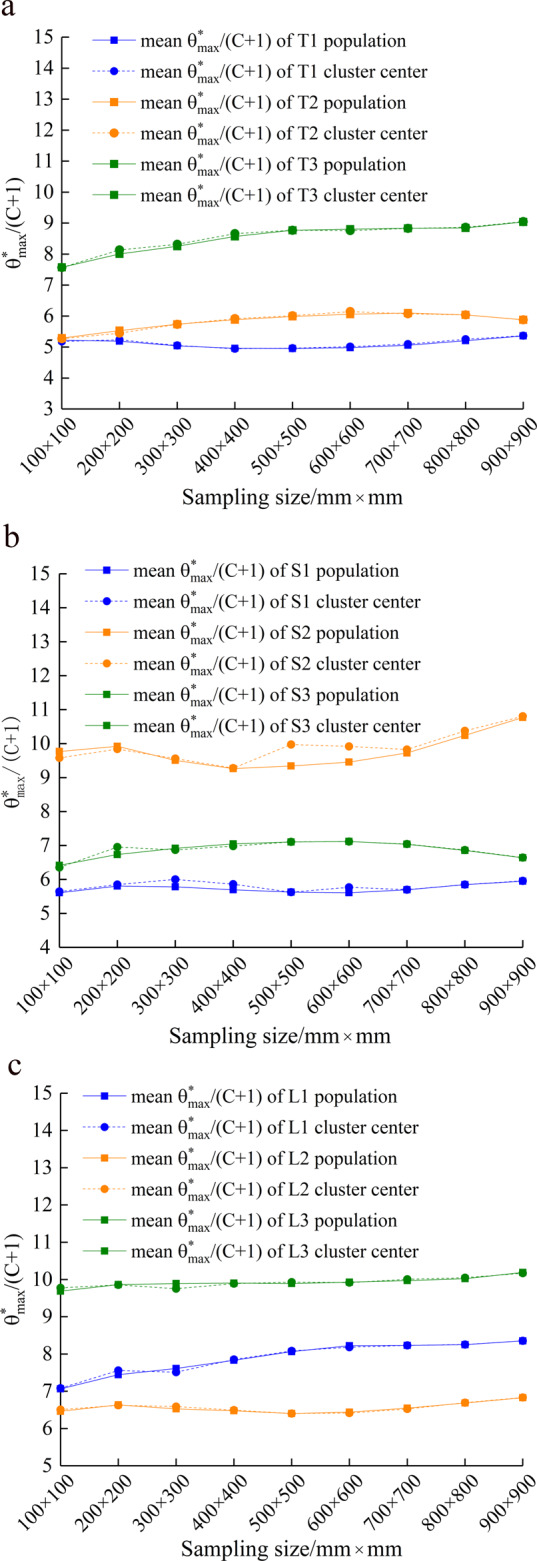
Evaluation of the cluster results. (**a**) tuff; (**b**) sandstone; (**c**) limestone.

Sampling of the joint model requires that the selected sample be representative of the undulating surface morphology under a specimen size. Considering that the proposed sampling method is based on the roughness statistics, Fig. 6 shows the variation of mean ${\theta }_{\max }^{\ast }/(C+1)$ of the cluster centers (${L}_{c}$) and the mean ${\theta }_{\max }^{\ast }/(C+1)$ of population (${L}_{p}$). The relative error $\delta$ is calculated as follows:

8 $$\delta =\frac{|{L}_{p}-{L}_{c}|}{{L}_{p}}\times 100 \% .$$

The obtained results have good coherence, in which the maximum error is 6.78% (see Supplementary Table S2). Therefore, the sampling results obtained by the K-medoids clustering algorithm can meet the test accuracy requirements.

## Comparison and Discussion

### Representative verification of interlayer cluster centers

**Figure 7:**
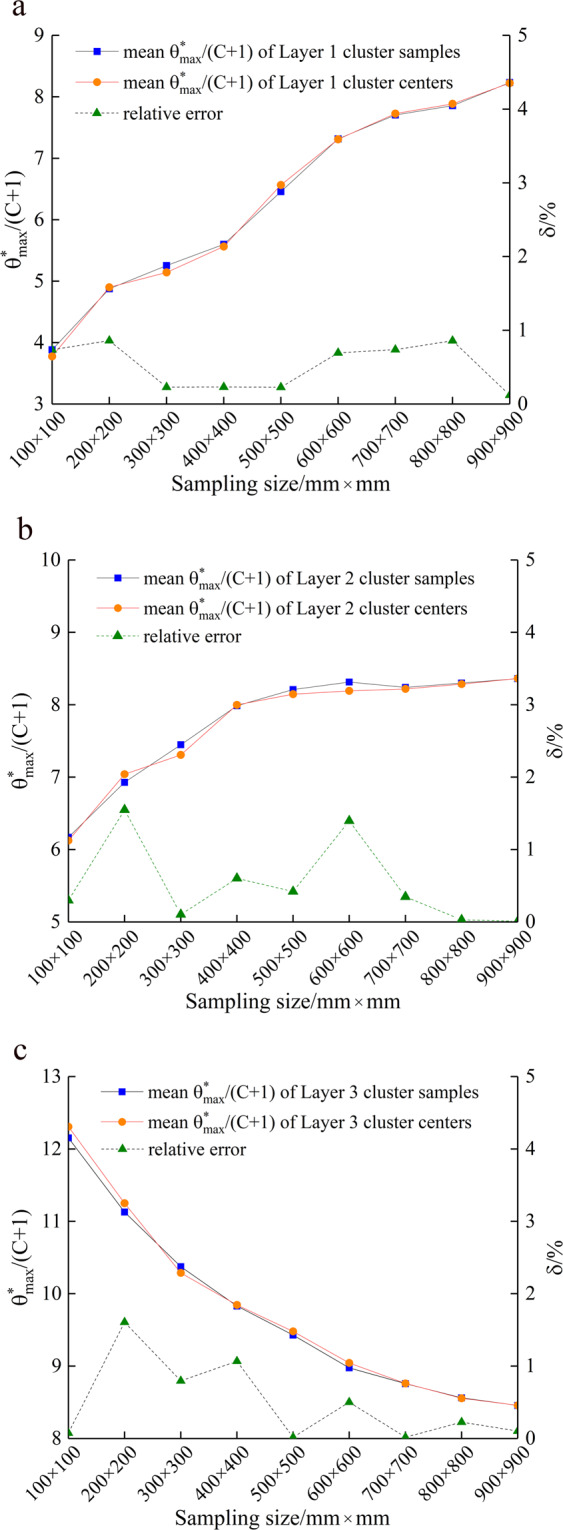
Evaluation of the interlayer cluster results.

From the process of K-medoids stratified sampling, the whole representativeness of the sample depends on the representativeness of the interlayer cluster centers. To demonstrate the representativeness of the cluster centers in each layer, we take the T1 joint as the research object and compare the mean ${\theta }_{\max }^{\ast }/(C+1)$ of the cluster samples of three layers and the mean ${\theta }_{\max }^{\ast }/(C+1)$ of the cluster centers (Fig. 7). The results show that each interlayer cluster center is almost identical to the average of the cluster samples. This finding indicates that the K-medoids clustering algorithm can ensure the consistency of the cluster results, which is the biggest feature and advantage of this method in representative sampling.

### Comparison with different sampling method

**Figure 8:**
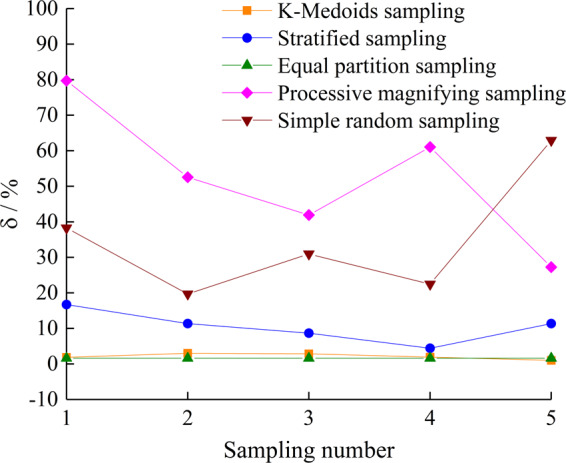
Sample distribution of different sampling methods.

To compare with the different sampling methods, we take the sampling size at 100 mm × 100 mm of the T1 joint as an example. First, the progressive coverage statistical method is used to get population samples, and then the roughness ${\theta }_{\max }^{\ast }/(C+1)$ of all samples are calculated to obtain population mean. Second, five repeated samplings are performed with Simple random sampling, Processive magnifying sampling, Equal partition sampling, Stratified sampling and K-medoids sampling methods, and then the roughness ${\theta }_{\max }^{\ast }/(C+1)$ of samples are calculated to obtain sample mean. Finally, the relative error between the sample mean of different sampling times and the population mean is calculated, as shown in Fig. 8. Although the results show that the relative error using the Equal partition sampling method is more constant and smaller, the large sample capacity will increase the burden for successive test. Therefore, the K-medoids sampling method with relative errors basically stabilized below 5% and appropriate sample capacity is the best choice for the representative sampling of joint surface morphology.

## Conclusions

A new representative sampling method for rock joint surfaces presented in this paper, the major summaries and conclusions are as follows:

The performance of four traditional sampling methods that are frequently used in the joint model test, namely, the simple random sampling method, processive magnifying sampling method, equal-partition sampling method, and stratified sampling method, are investigated in representative sampling. The results show that the conventional sampling methods have different drawbacks in the sampling of series size joint samples, and their performances do not involve the representative characterization of large-sized ones.

A new sampling method that combines the progressive coverage statistical and K-medoids stratified sampling methods is proposed to achieve an intelligent representative sampling for rock joints. The reasonably allocated samples of each sampling size are selected, and the roughness representativeness of the samples is quantitatively evaluated on the basis of the statistical analysis of the ${\theta }_{\max }^{\ast }/(C+1)$ values. The relative errors between the mean ${\theta }_{\max }^{\ast }/(C+1)$ of the samples and the mean ${\theta }_{\max }^{\ast }/(C+1)$ of the population are almost entirely below 5%. This finding indicates that the new sampling method can effectively provide representative joint samples for the joint model tests.

The representative verification of the interlayer cluster centers is carried out. The results show that the K-medoids clustering algorithm can effectively achieve a reasonable allocation of cluster centers and ensures the representativeness of the sampled samples. In addition, in accordance with the comparison with the traditional stratified sampling method, the K-medoids clustering algorithm enables a stable representative sampling of joint samples.

To verify the mechanical reliability of the selected samples, the comparative analysis of the mechanics need be further studied. Furthermore, the scale effect of shear behavior based on the representative samples will be investigated via direct shear tests in the future.

## Supplementary information

Supplementary information.
