# Supplementary material for: A New Representative Sampling Method for Series Size Rock Joint Surfaces
Source: Sci Rep. 2020 Jun 4;10:9129. doi: 10.1038/s41598-020-66047-0 (PMC7272448; doi:10.1038/s41598-020-66047-0)
Supplement: Supplementary file 1 — Supplementary information. [file 41598_2020_66047_MOESM1_ESM.pdf]

Supplementary Information

Title: A New Representative Sampling Method for Series Size Rock Joint Surfaces

Authors: Man Huang[a,b]*, Chenjie Hong[a], Chengrong Ma[a], Zhanyou Luo[c], Shigui Du[a], Fei Yang[a]

[a]*Department of Civil Engineering, Shaoxing University, 508 Huancheng West Road, Shaoxing 312000, Zhejiang, China*

[b]*Department of Civil and Environmental Engineering, Louisiana State University, 3255 Patrick F. Taylor hall, Baton Rouge, Louisiana 70803, American*

[c]*Geotechnical Engineering Institute, Zhejiang University of Science and Technology, 318 Liuhe Road, Hangzhou 310023, Zhejiang, China*

* Corresponding author: Man Huang

Tel.: +86 13615752267

E-mail address: hmcadx@126.com

Table Caption

Table S1 Statistical values of $\theta^*_{\max}/(C+1)$ in nine sampling sizes.

Table S2 Relative error analysis of nine joint sampling results.

Table S1 Statistical values of $\theta^*_{\max}/(C+1)$ in nine sampling sizes.

Original joint	Sampling size (mm × mm)	$\theta^*_{\max}/(C+1)$					n	K		
		S^2_{0-25}	S^2_{25-75}	S^2_{75-100}	S^2_{0-100}	$\bar{\gamma}$		Layer 1	Layer 2	Layer 3
T1	100 × 100	0.077	0.701	2.315	-	5.218	6	2	4	2
	200 × 200	0.093	0.602	0.202	-	5.189	3	1	2	1
	300 × 300	0.065	0.441	0.065	-	5.041	2	1	2	1
	400 × 400	0.025	0.262	0.062	-	4.959	2	1	2	1
	500 × 500	0.009	0.128	0.075	-	4.951	1	1	2	1
	600 × 600	0.014	0.061	0.057	-	4.980	1	1	2	1
	700 × 700	0.003	0.035	0.015	-	5.059	1	1	2	1
	800 × 800	0.003	0.010	0.004	-	5.206	1	1	2	1
	900 × 900	-	-	-	0.076	5.361	1	1		
S1	100 × 100	0.112	0.933	4.970	-	5.608	9	3	6	3
	200 × 200	0.171	0.479	3.014	-	5.805	6	2	4	2
	300 × 300	0.269	0.281	1.665	-	5.784	4	1	2	1
	400 × 400	0.080	0.204	0.714	-	5.697	2	1	2	1
	500 × 500	0.014	0.111	0.400	-	5.627	1	1	2	1
	600 × 600	0.018	0.061	0.192	-	5.611	1	1	2	1
	700 × 700	0.020	0.034	0.069	-	5.695	1	1	2	1
	800 × 800	0.021	0.004	0.015	-	5.847	1	1	2	1
	900 × 900	-	-	-	0.090	5.949	1	1		
L1	100 × 100	0.578	1.030	16.786	-	7.067	15	4	8	4
	200 × 200	0.115	1.094	2.228	-	7.444	4	1	2	1
	300 × 300	0.137	0.706	0.463	-	7.611	2	1	2	1
	400 × 400	0.275	0.646	0.093	-	7.833	2	1	2	1
	500 × 500	0.356	0.387	0.050	-	8.063	1	1	2	1
	600 × 600	0.051	0.113	0.013	-	8.222	1	1	2	1
	700 × 700	0.020	0.034	0.007	-	8.231	1	1	2	1
	800 × 800	0.008	0.021	0.002	-	8.251	1	1	2	1
	900 × 900	-	-	-	0.089	8.352	1	1		

Table S2 Relative error analysis of nine joint sampling results.

Original joint	Sampling size (mm × mm)	δ/%	Original joint	Sampling size (mm × mm)	δ/%	Original joint	Sampling size (mm × mm)	δ/%
	100 × 100	0.737		100 × 100	0.298		100 × 100	0.077
	200 × 200	0.860		200 × 200	1.549		200 × 200	1.607
	300 × 300	0.229		300 × 300	0.103		300 × 300	0.797
	400 × 400	0.230		400 × 400	0.603		400 × 400	1.068
T1	500 × 500	0.228	T2	500 × 500	0.421	T3	500 × 500	0.016
	600 × 600	0.695		600 × 600	1.396		600 × 600	0.502
	700 × 700	0.738		700 × 700	0.348		700 × 700	0.018
	800 × 800	0.858		800 × 800	0.030		800 × 800	0.226
	900 × 900	0.121		900 × 900	0.010		900 × 900	0.103
	100 × 100	0.607		100 × 100	1.948		100 × 100	0.971
	200 × 200	0.732		200 × 200	0.813		200 × 200	3.275
	300 × 300	3.740		300 × 300	0.551		300 × 300	0.694
	400 × 400	2.899		400 × 400	0.172		400 × 400	0.937
S1	500 × 500	0.012	S2	500 × 500	6.784	S3	500 × 500	0.035
	600 × 600	2.830		600 × 600	4.874		600 × 600	0.071
	700 × 700	0.056		700 × 700	1.053		700 × 700	0.061
	800 × 800	0.059		800 × 800	1.301		800 × 800	0.215
	900 × 900	0.168		900 × 900	0.340		900 × 900	0.040
	100 × 100	0.233		100 × 100	0.718		100 × 100	0.899
	200 × 200	1.534		200 × 200	0.159		200 × 200	0.100
	300 × 300	1.314		300 × 300	0.945		300 × 300	1.398
	400 × 400	0.241		400 × 400	0.283		400 × 400	0.153
L1	500 × 500	0.262	L2	500 × 500	0.010	L3	500 × 500	0.361
	600 × 600	0.461		600 × 600	0.367		600 × 600	0.105
	700 × 700	0.005		700 × 700	0.310		700 × 700	0.350
	800 × 800	0.025		800 × 800	0.131		800 × 800	0.253
	900 × 900	0.001		900 × 900	0.076		900 × 900	0.190

Figure Caption

Fig. S1 Diagram of the progressive coverage statistical method. (a) small unit sample; (b) medium unit sample; (c) large unit sample.

Fig. S2 3D morphology of nine rock joints. T1-T3 = tuff; S1-S3 = sandstone; L1-L3 = limestone.

Fig. S3 Frequency histogram of different sample sizes. (a) 100 mm × 100 mm; (b) 200 mm × 200 mm; (c) 300 mm × 300 mm; (d) 400 mm × 400 mm; (e) 500 mm × 500 mm; (f) 600 mm × 600 mm; (g) 700 mm × 700 mm; (h) 800 mm × 800 mm; (i) 900 mm × 900 mm.

Fig. S4 Cluster results of different sampling sizes. (a) 100 mm × 100 mm; (b) 200 mm × 200 mm; (c) 300 mm × 300 mm; (d) 400 mm × 400 mm; (e) 500 mm × 500 mm; (f) 600 mm × 600 mm; (g) 700 mm × 700 mm; (h) 800 mm × 800 mm; (i) 900 mm × 900 mm.

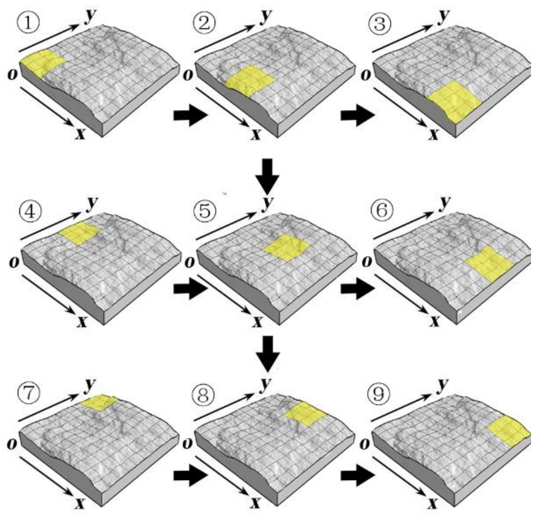

a

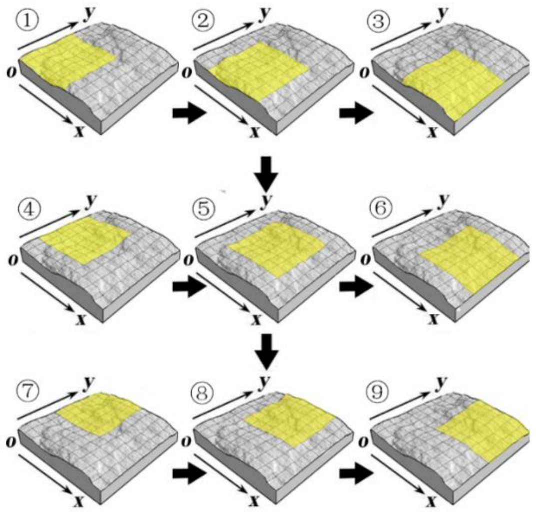

b

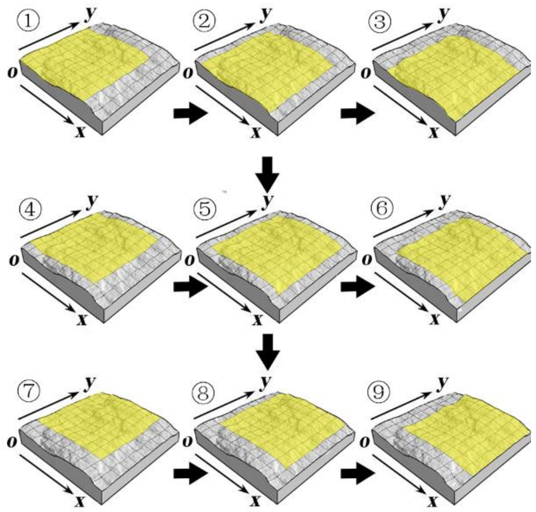

c

Fig. S1 Diagram of the progressive coverage statistical method. (a) small unit sample; (b) medium unit sample; (c) large unit sample.

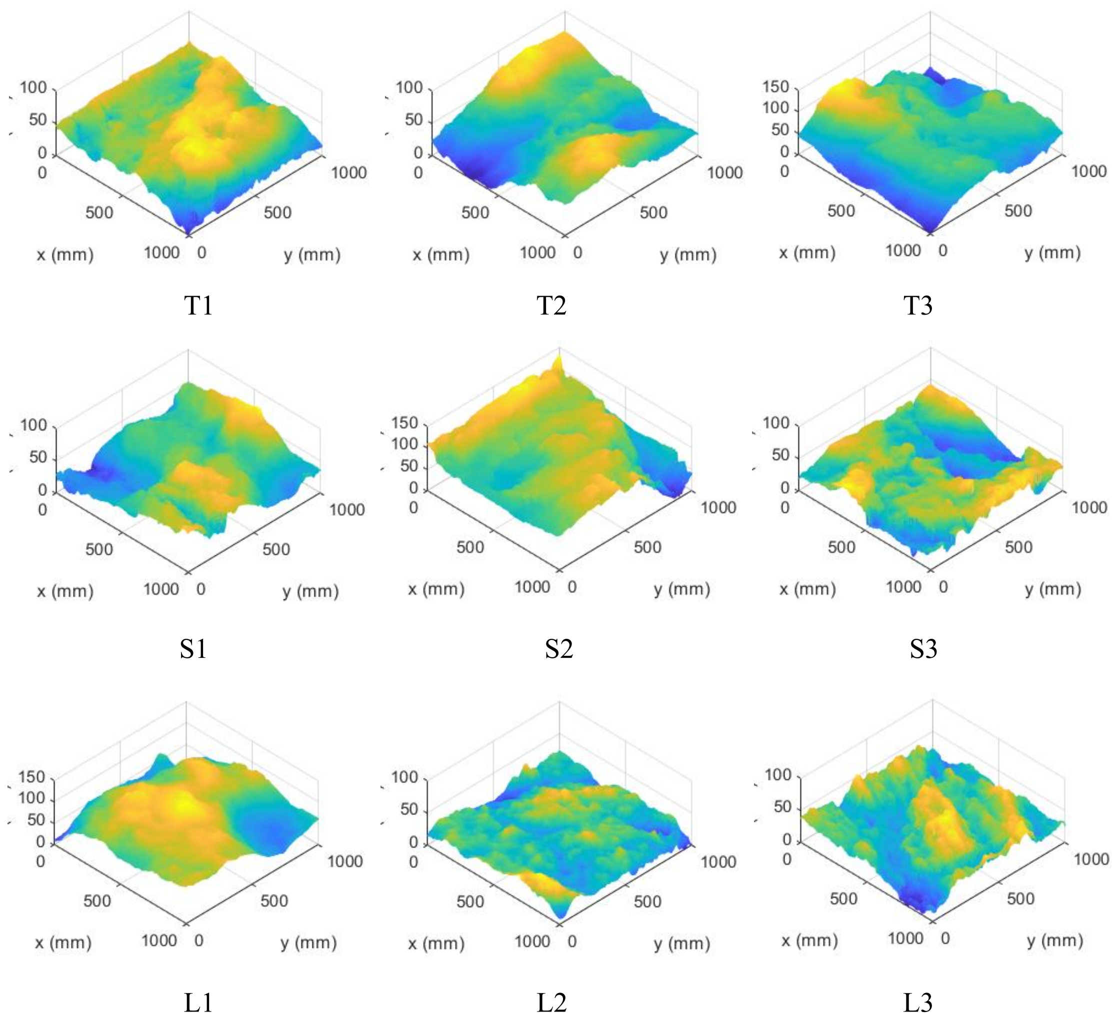

Fig. S2 3D morphology of nine rock joints. T1-T3 = tuff; S1-S3 = sandstone; L1-L3 = limestone.

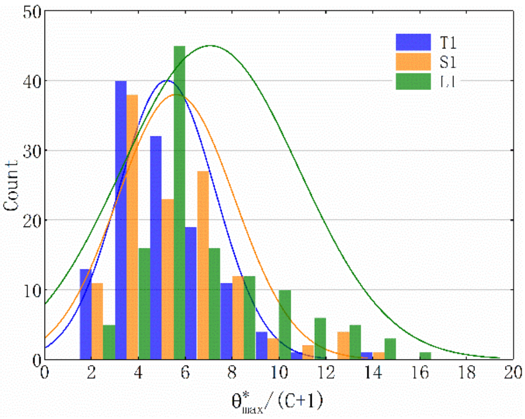
T1
S1
L1
Count
θ*max / (C+1)

a

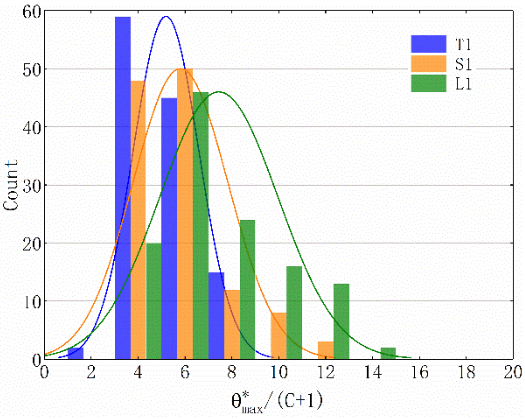
T1
S1
L1
Count
θ*max / (C+1)

b

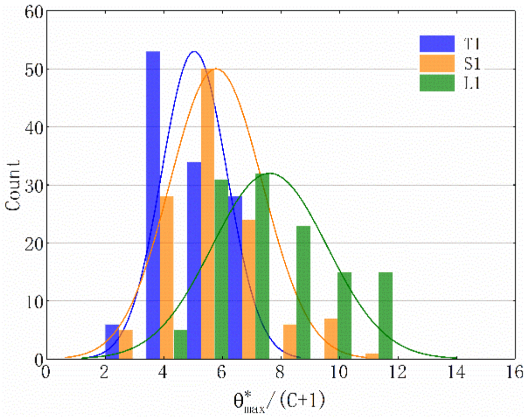
T1
S1
L1
Count
θ*max / (C+1)

c

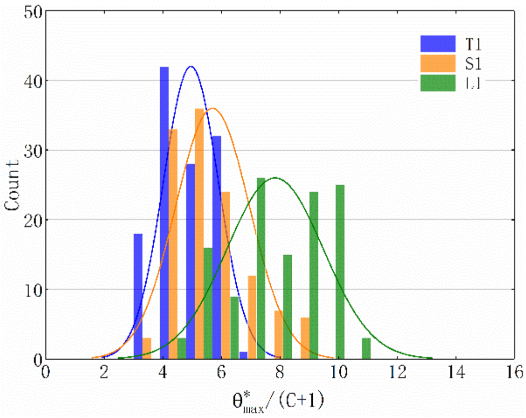
T1
S1
L1
Count
θ*max / (C+1)

d

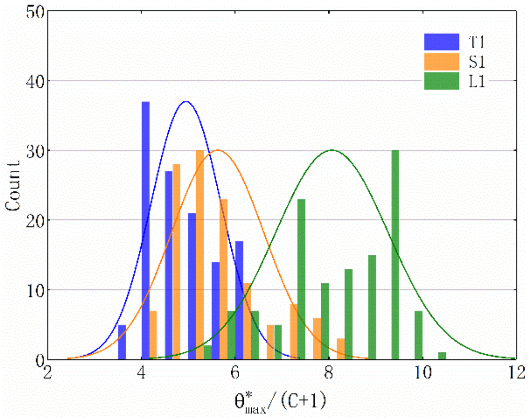
T1
S1
L1
Count
θ*max / (C+1)

e

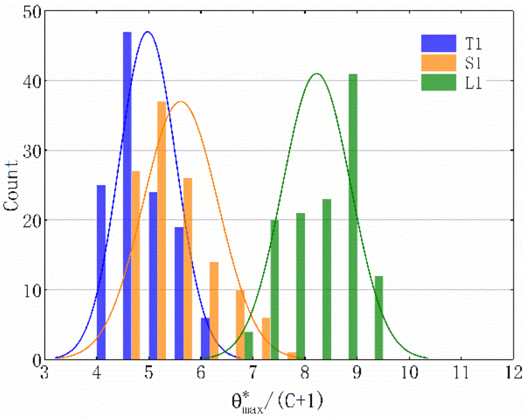
T1
S1
L1
Count
θ*max / (C+1)

f

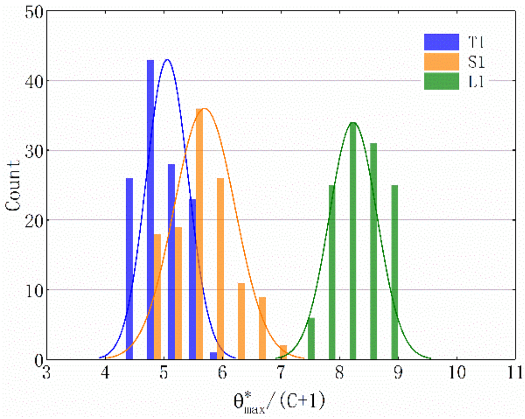

g

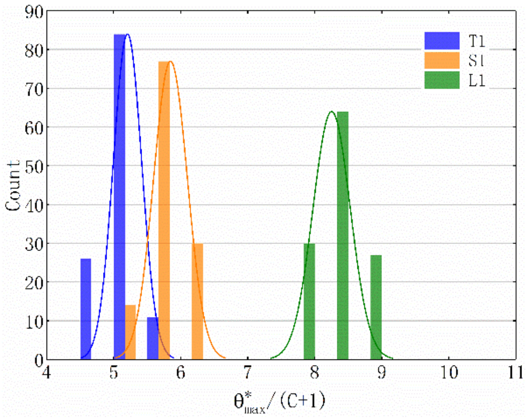

h

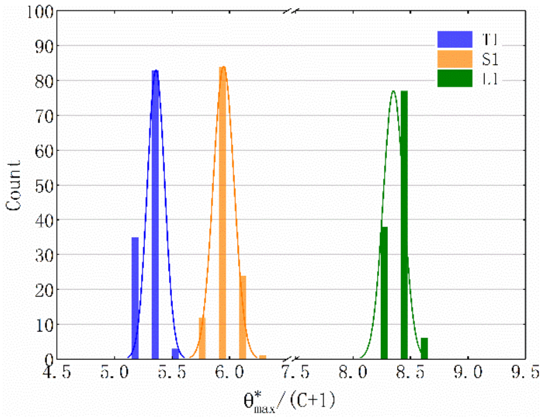

i

Fig. S3 Frequency histogram of different sample sizes. (a) 100 mm × 100 mm; (b) 200 mm × 200 mm; (c) 300 mm × 300 mm; (d) 400 mm × 400 mm; (e) 500 mm × 500 mm; (f) 600 mm × 600 mm; (g) 700 mm × 700 mm; (h) 800 mm × 800 mm; (i) 900 mm × 900 mm.

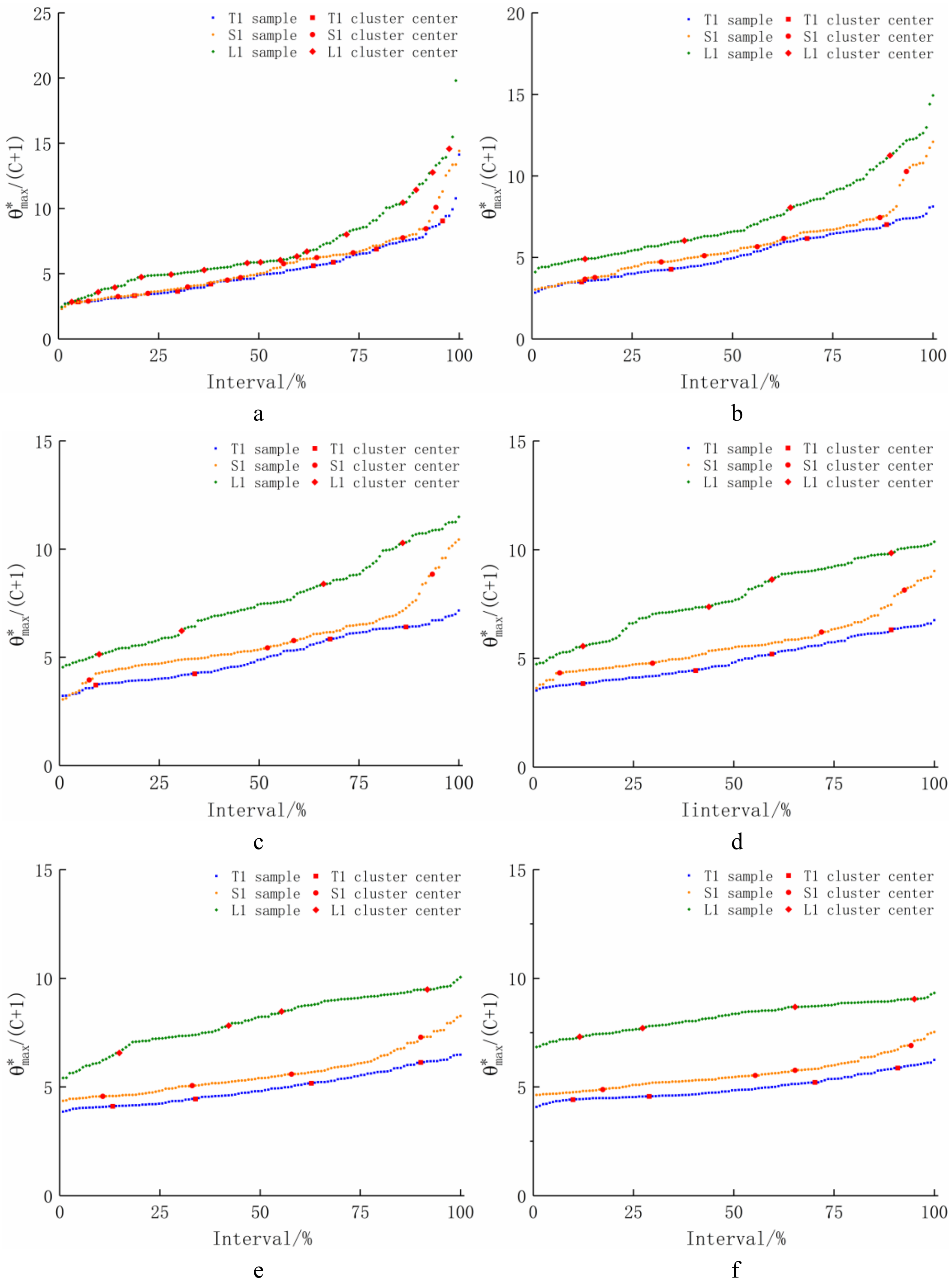
T1 sample
T1 cluster center
S1 sample
S1 cluster center
L1 sample
L1 cluster center
θ*max/(C+1)
Interval/%
Iinterval/%
a
b
c
d
e
f

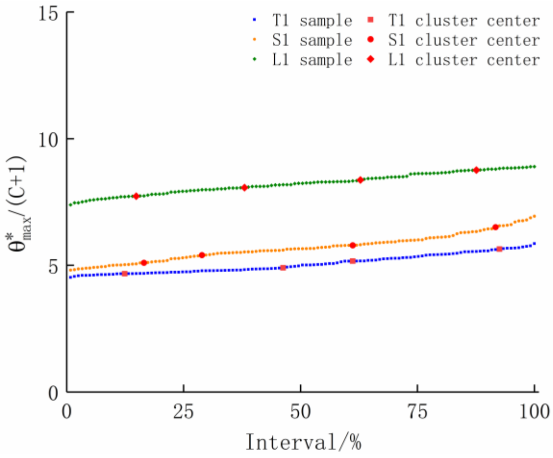

g

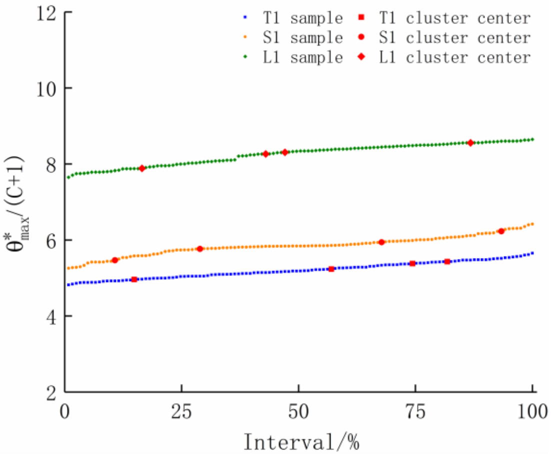

h

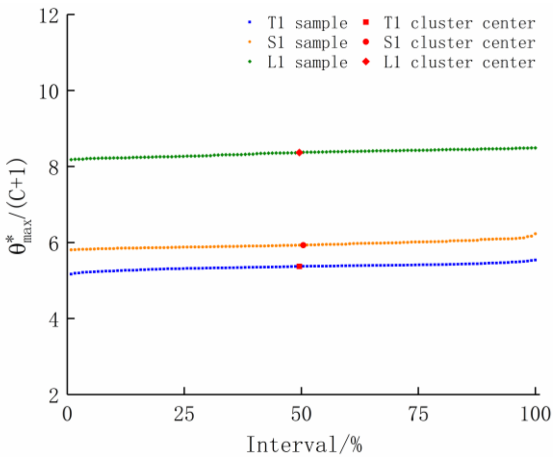

i

Fig. S4 Cluster results of different sampling sizes. (a) 100 mm × 100 mm; (b) 200 mm × 200 mm; (c) 300 mm × 300 mm; (d) 400 mm × 400 mm; (e) 500 mm × 500 mm; (f) 600 mm × 600 mm; (g) 700 mm × 700 mm; (h) 800 mm × 800 mm; (i) 900 mm × 900 mm.
